# Defect‐Engineered MOF/COF S‐Scheme Heterojunction With Dual‐Channel Charge Transport for Ultraefficient Solar‐Powered Heavy Metal Remediation

**DOI:** 10.1002/advs.202520433

**Published:** 2026-01-15

**Authors:** Yuqian Zhong, Xinpeng Wang, Weiqun Shi, Liyong Yuan

**Affiliations:** ^1^ Institute of High Energy Physics Chinese Academy of Sciences Beijing P. R. China; ^2^ School of Resource, Environment and Material Guangxi University Nanning P. R. China; ^3^ School of Mechanical Engineering Shanghai Jiao Tong University Shanghai P. R. China

**Keywords:** heavy metal remediation, oxygen‐vacancy, photocatalysis, S‐scheme

## Abstract

Rapid recombination of photogenerated carriers severely limits the photocatalytic performance of conventional semiconductor photocatalysts, while conventional heterojunctions generally suffer from inefficient charge separation and sluggish interfacial kinetics due to poor lattice matching and unidirectional recombination. Herein, we break through these limitations by constructing an oxygen vacancies (OVs)‐mediated S‐scheme via covalent bridging between a metal–organic framework (MOF) and a covalent organic framework (COF), coupled with vacuum‐induced OVs engineering. This novel architecture not only preserves the strong redox potentials of the constituent materials but also introduces dual‐channel charge transport pathways significantly enhancing carrier separation. Femtosecond transient absorption spectroscopy (fs‐TAS) reveals that the OVs‐induced trap states extend the carrier lifetime to 278 ps—2.5 times longer than the parent materials. The optimized catalyst achieves exceptional removal efficiencies for multiple heavy metal ions (Cu⁺, ReO_4_^−^, MoO_4_^2^^−^, MnO_4_^−^, Cr_2_O_7_^2^^−^, and UO_2_^2^⁺), with UO_2_^2^⁺ removal rates 8.8 and 17.1 times higher than those of the pristine MOF and COF, respectively. This work presents a universal “defect‐mediated dual transport” strategy, offering new insights into solar‐driven environmental purification and energy conversion.

## Graphical abstract

Covalent bond MOF/COF S‐scheme which generated via vacuum‐induced oxygen vacancies are report. OVs at the interface can trigger a dual‐channel charge transport mechanism, significantly enhancing catalytic performance. This work not only designs a high‐performance photocatalysts for environmental remediation, but also induces a new concept of “defect engineering for charge transport optimization”, providing a groundbreaking approach to controlling charge dynamics in next generation photocatalytic systems.

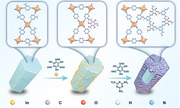

## 1 Introduction

The escalating global demand for energy and the concurrent environmental pollution crisis underscore the urgent need for sustainable technologies that integrate clean energy utilization with effective environmental remediation [1]. Photocatalysis, which harnesses solar energy to drive redox reactions for pollutant degradation, has emerged as a promising dual‐purpose solution [2]. However, conventional semiconductor photocatalysts are limited by the rapid recombination of photogenerated electron‐hole pairs, leading to low quantum efficiency and constrained catalytic performance [3]. In recent years, S‐scheme have presented a novel avenue for enhancing photocatalytic activity [4, 5, 6]. These structures facilitate efficient spatial charge separation while maintaining high redox potentials of the charge carriers [7, 8]. Nevertheless, their overall performance remains hindered by a persistent bottleneck—a single, slow interfacial charge transport pathway that is still vulnerable to recombination losses.

Current research predominantly emphasizes modifications at the heterojunction interface. Wang et al. [9]. For example, implemented a synergistic strategy combining crystal plane regulation of ZIF‐67 with the construction of an “Fe‐N‐Co” bridge Z‐type heterojunction, which significantly enhanced nitrogen activation and charge separation. This engineered system enabled highly efficient photocatalytic ammonia synthesis, reaching a production rate of 33.2 mmolg^−1^h^−1^. Li et al. [10]. developed a unique core‐shell structure am@TiO_2_ by introducing an amorphous TiO_x_ layer onto crystalline TiO_2_ through an oxidative etching method. The resulting amorphous surface, enriched with ─OH group, facilitated the uniform deposition of nanoscale IrO_x_ co‐catalysts. This modification led to an oxygen evolution performance 14 times higher than the unmodified TiO_2_ photocatalyst. However, this morphology tuning and surface engineering strategy often overlooking the fundamental role of “bulk phase” charge transport behavior [11]. Although these strategies offer incremental improvements, they fall short in addressing the core issue of charge recombination, thereby limiting breakthroughs in photocatalytic efficiency.

To overcome this challenge, the present study proposes a paradigm‐shifting strategy: a defect‐mediated dual‐channel charge transport mechanism. Specifically, a controlled concentration of oxygen vacancies (OVs) is introduced into metal‐organic framework/covalent organic framework (MOF/COF) heterojunctions [12, 13] through a vacuum thermal induction process [14, 15, 16], which also caused the two organic‐framework contact to bond via covalent bridge. These vacancies, traditionally viewed as charge traps detrimental to performance [17, 18, 19], are here redefined as an independent and efficient second electron transport channel within the bulk phase, operating in parallel with the heterojunction interface. The first channel, driven by the built‐in electric field of the S‐scheme heterojunction [20, 21], ensures directional separation of photogenerated carriers. The second channel, enabled by OVs‐induced defect states, acts as an electronic “viaduct”, facilitating fast and efficient electron migration. This dual‐channel synergy effectively mitigates recombination losses and surpasses the efficiency ceiling of conventional single‐path transport systems.

Experimental validation demonstrated that the newly developed catalysts achieved exceptional photocatalytic performance, with UO_2_^2+^ removal rates enhanced by factors of 8.8 and 17.1 compared to the pristine material. Moreover, the system exhibited broad‐spectrum removal capabilities for various heavy metal ions, such as Cr_2_O_7_^2−^ and MnO_4_^−^. Transient Adsorption Spectroscopy (fs‐TAS) confirmed the formation of additional transport pathways using OVs‐mediated electron capture states. This study not only introduces a high‐performance material platform for solar‐driven environmental remediation but also establishes the innovative concept of “defect engineering for charge transport optimization”. This conceptual advance offers a transformative approach to controlling charge dynamics in next‐generation photocatalytic systems.

## 2 Results and Discussion

### 2.1 Morphological and Structural Characterizations

MOF/COF composites are named MIL@COF, the MIL@COF were constructed through covalent bonding, wherein aldehyde‐functionalized MIL‐68 (MIL‐CHO) served as a crystalline seed to direct the epitaxial growth of TpTt‐COF. Depending on the loading amount of TpTt‐COF, we respectively named the composites as MIL@COF_x_ (MIL@COF_0.5_, MIL@COF_1.0,_ and MIL@COF_2.0_). At the heterojunction interface, amide bonds formed between MIL‐68 and TpTt‐COF, acting as covalent bridges that integrate the hierarchical porosity of the MIL‐68 with the structural regularity of TpTt‐COF. Unlike conventional inorganic semiconductor heterojunctions, which rely on weak van der Waals interactions, the covalently bonded interface in MIL@COFx ensures enhanced structural stability and facilitates efficient charge carrier mobility across the framework. This synergy leverages the inherent advantages of both porous materials, enabling precise control over electron transport pathways.

**FIGURE 1:**
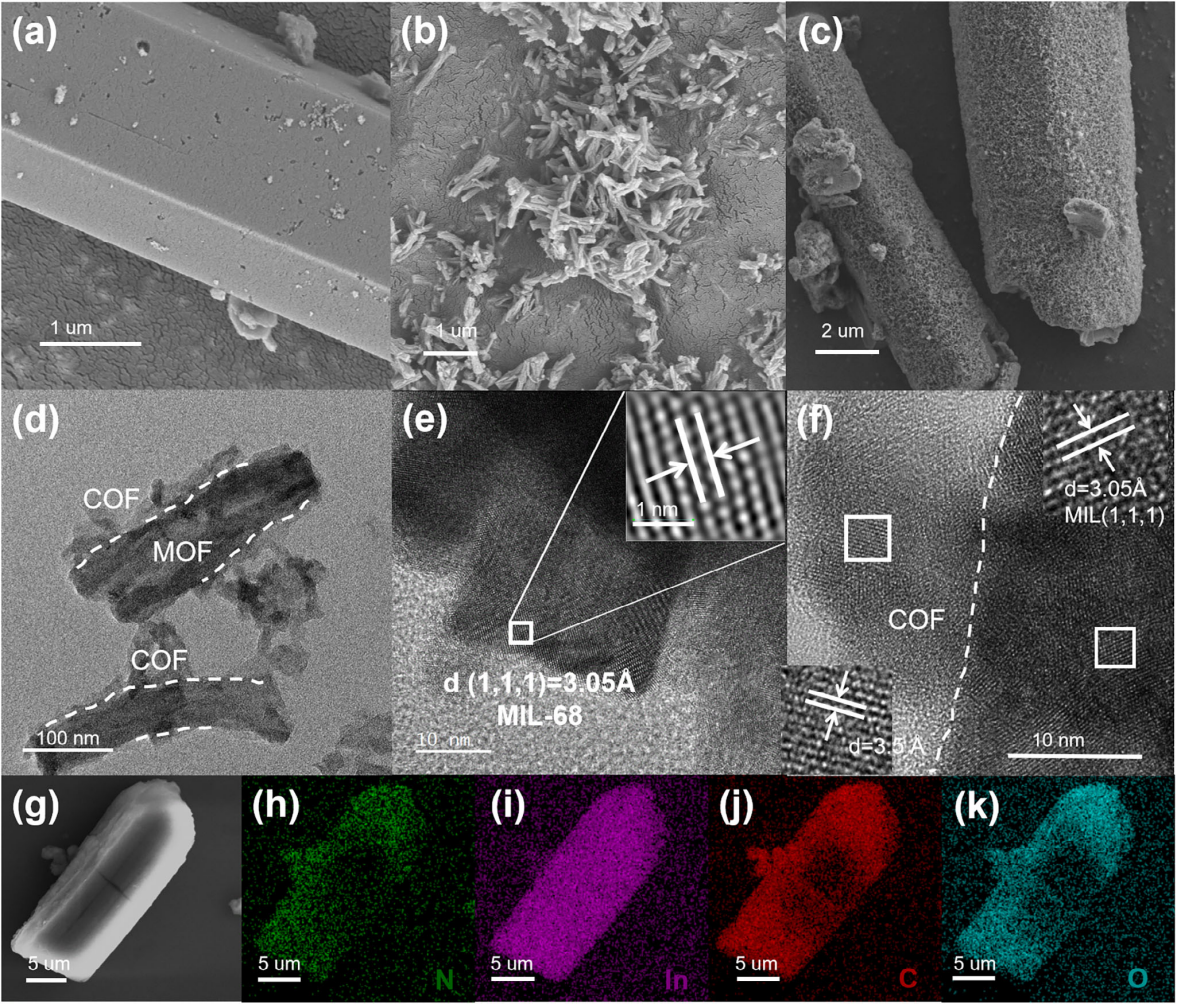
(a) SEM images of MIL‐68, (b) TpTt‐COF, and (c) MIL@COF; (d) HRTEM images of MIL@COF; (e,f) determined lattice space with lattice fringes for IFFT and HRTEM; (g) SEM; h∼k) corresponding EDS elemental mapping images of MIL@COF composites.

As shown in the scanning electron microscopy (SEM) images (Figure 1a), the pristine MIL‐68 exhibits a smooth surface (Figure S1), whereas MIL@COF displays a textured, rough morphology resembling the fibrous structure of pure TpTt‐COF (Figure 1b, Figure S2). This contrast confirms the successful coating of TpTt‐COF onto the MIL‐68 surface, forming a core‐shell architecture. The size of MIL@COF is significantly smaller than MIL‐68, that is due to the fragmentation caused by the processing. High‐resolution transmission electron microscopy (HRTEM) further verifies this hierarchical structure. For instance, in MIL@COF (Figure 1c), the TpTt‐COF shell appears as a ∼30 nm‐thick layer with a porous, flaky morphology, consistent with the intrinsic hollow nature of the COF. Lattice fringes in the core region correspond to MIL‐68, showing a d‐spacing of 2.52 Å along the {0,1,1} crystallographic plane (Figure 1d). Concurrently, TpTt‐COF shell exhibits periodic interlayer spacings of 3.5 Å (Figure 1e,f), characteristic of its layered stacking and high crystallinity [22]. Elemental mapping (Figure 1g–k) confirms the uniform distribution of indium (In), carbon (C), and oxygen (O) throughout the MIL@COF heterostructure, corroborating the homogeneous integration of both components.

**FIGURE 2:**
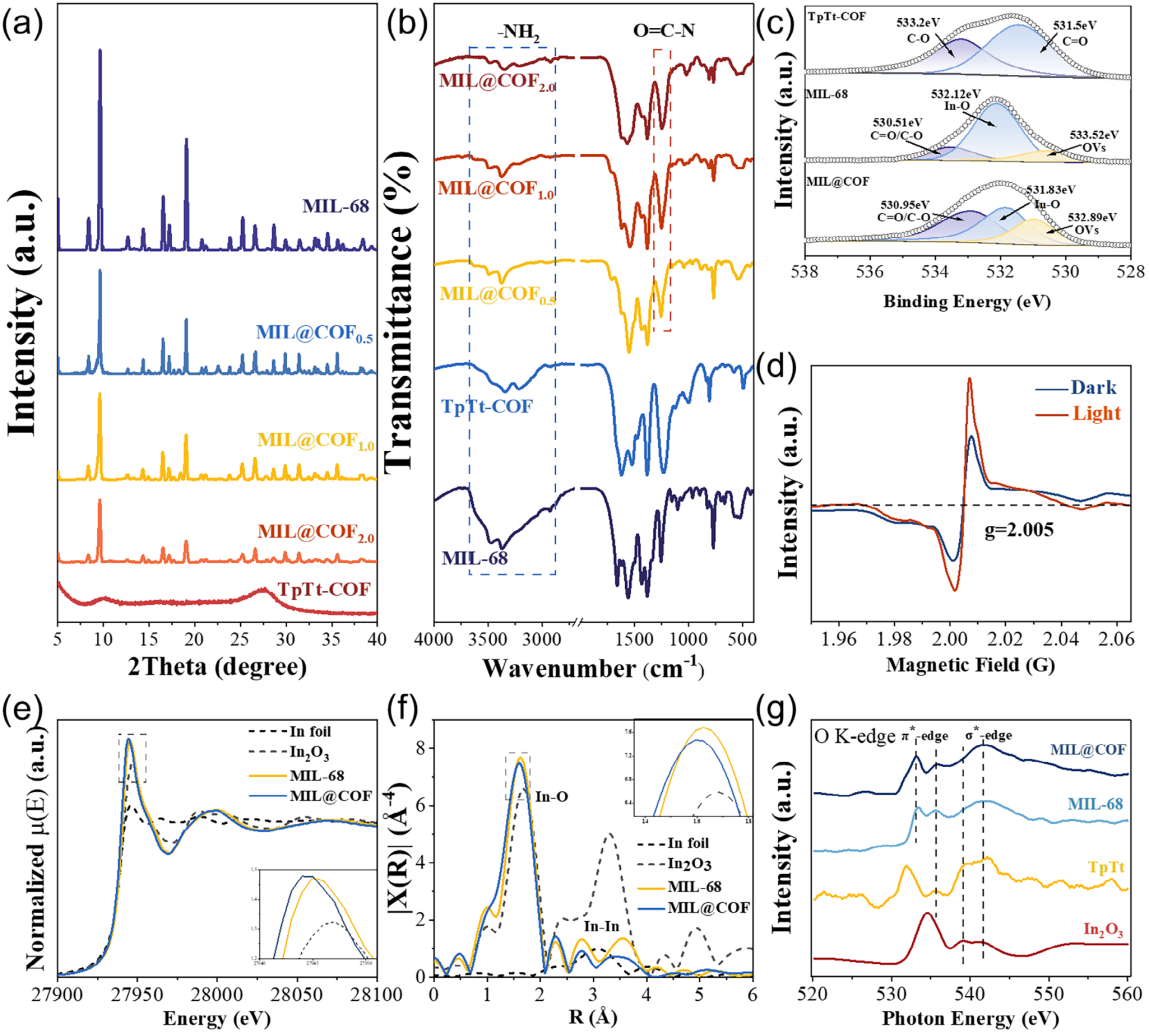
(a) Powder XRD patterns, (b) FT‐IR absorption spectra, (c) high‐resolution XPS spectra of O 1s, (d) EPR spectra of synthesized samples, In K‐edge (e) XANES spectra, (f) EXAFS spectra, and (g) XAS spectra of O K‐edge.

The crystalline of MIL‐CHO was also evaluated by powder X‐ray diffraction (PXRD, Figure S3). Its diffraction pattern closely matched that of the parent MIL‐68, confirming structural retention after functionalization [23]. Further PXRD analysis of TpTt‐COF and its MIL@COF composites revealed distinct structural features. TpTt‐COF exhibited two broadened diffraction peaks at 9.5° and 27.6° (2θ), indexed to the {220} and {001} crystallographic planes [22], respectively, with cell parameters a = 11.2 Å, b = 11.05 Å, and c = 6.98 Å. The reduced crystallinity of TpTt‐COF, compared to large‐pore COFs, arises from its small pore size (∼1.2 nm), which promotes denser π‐stacking and lattice distortions. According to Bragg's law (nλ = 2d sinθ), the smaller interlayer spacing (d) in TpTt‐COF shifts its primary diffraction peaks to higher angles (e.g., 27.6°), consistent with its compressed unit cell. In the MIL@COF composites, the intensity of MIL‐68's characteristic diffraction peaks progressively diminished with increasing COF loading (Figure 2a). This trend reflects the dilution of the MOF phase within the composite: as COF content rises, the volume fraction of MOF crystallites interacting with X‐rays decreases, reducing constructive interference and peak intensity.

The bonding modes of the materials were characterized using Fourier transform infrared spectroscopy (FT‐IR). First, the IR spectra of the MIL‐CHO intermediates are compared (Figure S4). The ‐N‐H vibrational peaks of MIL‐CHO are significantly attenuated compared to those of MIL‐68. This attenuation was attributed to the involvement of some ‐NH_2_ groups in the condensation reaction with the ─CHO group on the Tp ligand, as evidenced by the appearance of the ─CHO stretching peaks (2720 to 2820 cm^−1^). The IR spectra of the parent MIL‐68 are compared with those of the MIL@COFx complexes with varying doping levels (Figure 2b). It was observed that the ─N─H stretching vibrational absorption peaks (3500 to 3200 cm^−1^) [24] gradually diminished, while new peaks corresponding to ─C─N‐ bonds appeared. This suggests that the amino groups on MIL‐68 successfully formed covalent bonds with the aldehyde groups on Tp. When comparing the absorption peaks of ─C─N─ stretching vibrations in the range from 1360 to 1200 cm^−1^ between the parent TpTt‐COF and the MIL@COFx complexes with different doping levels [25], it was found that the ─C─N‐ peaks are gradually redshifted toward lower wave numbers as the doping amount of COF increased [26]. This phenomenon can be attributed to differences in the molecular environments of the carbon and nitrogen single bonds in COF, as well as those in MIL and Tp. The peak position in this region is also influenced by other functional groups and the overall molecular structure, leading to a slight displacement of the peak.

To elucidate the presence of OVs in MIL@COF, characterizations were performed using X‐ray photoelectron spectroscopy (XPS) and electron spin resonance (ESR) spectroscopy. The high‐resolution XPS spectrum of O 1s is shown in Figure 2c. The peaks locate at 532.89, 531.83, and 530.95 eV correspond to OVs, In‐O coordination bonds, and C═O/C─O bonds, respectively [27]. A comparison between pristine MIL‐68 and the MIL@COF heterostructure reveals a substantial increase in both the peak area and intensity associate with OVs in MIL@COF. The peaks at 533.2 and 531.5 eV in TpTt‐COF are attributed to the C─O bond without tautomerism, and the C═O bond in Tp, respectively, and no signal of OVs was detected [28]. By integrating the corresponding peak areas from the O 1s spectra, we calculated the percentage distribution of various chemical states of oxygen (Figure S5). The proportion of OVs increase notably from 15% in MIL‐68% to 23% in MIL@COF, indicating the formation of abundant OVs upon constructing the composites. The negative shift (E = −0.29 eV) in the binding energy of the In─O bonds is observed from the In 3d spectra indicates electron redistribution due to OVs. This shift provides further evidence that partial breaking of In‐O bonds contributes directly to the formation and subsequent increase of OVs in MIL@COF. Figure 2d displays the electron spin resonance (ESR) spectra of MIL@COF, exhibiting a distinct OVs signal that is notably enhanced upon light irradiation.

K‐edge XANES spectra of In (Figure 2e) shows that the white line intensity of the prepared MIL@COF was slightly lower than that of the pristine MIL‐68. Weaker white line absorption peaks are associated with an increase in the electron density of the In 3d band in MIL@COF. After surface growth of TpTt, MIL@COF exhibits higher intensity peaks, revealing a lower average electron density and higher In oxidation state in MIL@COF compared to MIL‐68, strongly validating the loss of electrons from MIL‐68 in MIL@COF. The In K‐edge EXAFS spectra of MIL‐68 and MIL@COF are shown in Figure 2f. Fitting of the In K‐edge EXAFS curves shows that the decrease in the number of coordination sites in MIL@COF compared to MIL‐68 is related to the absence of oxygen atoms [29]. In addition, O K‐edge spectra are also used to demonstrate the presence and state of OVs in the material (Figure 2g), with peaks around 540 eV being attributed to the σ‐edge and peaks around 535 eV at the π‐edge in the O K‐edge spectra. Compared with the spectrum of MIL‐68, the peak width of the π‐edge absorption peaks of MIL@COF was increased, indicating that the material‐averaged metal‐oxygen bond is elongated and the coordination number is decreased [30]; meanwhile, the peaks are shifted to lower energies, and the two electrons left behind by vacancies partially compensate for the neighboring metal d orbitals, weakening the π‐bonding back‐bonding coupling of O‐In, which further suggests that the vacuum‐thermal‐induced synthesis method effectively improves the concentration of the OVs in the material [31].

### 2.2 Electronic Structure and Band Engineering

**FIGURE 3:**
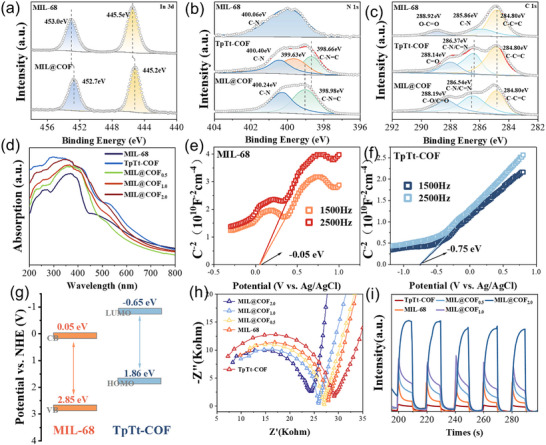
High‐resolution XPS spectra of (a) In 3d, (b) N 1s, and (c) C 1s before and after illumination. (d) UV‐vis diffuse absorption spectra. (e, f) Mott‐Schottky plots, and (g) energy‐band structure plots of the complexes. (h) electrochemical impedance spectra (EIS), (i) transient photocurrent densities.

XPS was further conducted to investigate the surface composition and chemical interactions in MIL@COF composites. The full XPS spectra of MIL‐68, TpTt‐COF, and MIL@COF are presented in Figure S6. The XPS analysis confirmed the presence of C, N, O, and In elements in the MIL@COF heterojunction, further validating the successful synthesis of the composite. Notably, the binding energy of the In 3d energy level in MIL@COF shifted toward a higher binding energy compared to pristin MIL‐68 (Figure 3a). This shift indicates that MIL‐68 loses electrons, leading to electron migration outward from the material. Additionally, the N 1s spectrum revealed that the diffraction peaks corresponding to the characteristic triazine functional group (C─N═C) in COF moved to a lower binding energy upon binding with MOF (Figure 3b). Similarly, the C═N/C─N peaks in the C 1s spectrum also shifted to a lower binding energy (Figure 3c), aligning with the previous findings and indicating an increase in electron cloud density around COF. This directional migration of electrons from MIL‐68 to TpTt‐COF leads to the formation of an effective built‐in electric field at the heterojunction interface [32]. The presence of this built‐in electric field facilitates the directional migration of photogenerated carriers during the catalytic process, which plays a crucial role in enhancing the photocatalytic activity of the material.

We investigated the possible factors contributing to the enhanced photocatalytic performance of MIL@COF from three perspectives: solar absorption, intrinsic physical properties, and energy band alignment. As shown in Figure 3d, we analyzed the UV‐vis diffuse reflectance absorption spectra of the parent MIL‐68, TpTt‐COF, and their composite MIL@TpTt. The results indicate that the light absorption edge of MIL‐68 is 507 nm, while that of TpTt extends to nearly 700 nm. Compared to MIL‐68 and TpTt‐COF, the UV absorption edges of MIL@TpTt composites with different doping levels exhibit a redshift, extending beyond 700 nm. This redshift confirms that the composites possess an expanded light absorption range. As shown in Figure S7, the band gaps (E_g_) of MIL‐68 and TpTt are calculated to be 2.80 and 2.56 eV by the Kubelka‐Munk function, respectively. To clearly elucidate the energy band structure of the materials, the band edge positions of MIL‐68 and TpTt‐COF were determined by analyzing Motty–Schottky plots. As shown in Figure 3e,f, both MIL‐68 and TpTt‐COF exhibit negative slopes in their Motty–Schottky plots, indicating their characteristics as typical n‐type semiconductors. The flat‐band potentials (Vfb) of MIL‐68 and TpTt‐COF are determined relative to the Ag/AgCl electrode. The Vfb of MIL‐68 and TpTt‐COF are determined to be −0.05 and −0.75 V (vs. Ag/AgCl), respectively. In addition, it is generally accepted that the CB minimum of n‐type semiconductors is approximately 0.1 eV more negative than their flat‐band potentials [33]. Therefore, the CB positions of MIL‐68 and TpTt‐COF were calculated to be approximately −0.15 and −0.85 V (vs. Ag/AgCl), respectively. Considering that the potential difference between the Ag/AgCl electrode and the standard hydrogen electrode (NHE) is approximately 0.2 V [34], CB potentials of MIL‐68 and TpTt‐COF relative to NHE were calculated to be approximately 0.05 and −0.65 V, respectively. Based on the bandgap values obtained from UV‐vis measurements, VB potentials of MIL‐68 and TpTt‐COF relative to NHE were calculated to be approximately 2.85 and 1.86 V, respectively. The resulting band structures of both materials are illustrated schematically in Figure 3g.

Furthermore, the transfer and separation efficiency of photogenerated carriers is a critical factor in assessing photocatalytic performance. As shown in the electrochemical impedance spectroscopy (EIS) curves in Figure 3h, MIL@COF exhibits the lowest charge transfer resistance compared to the parent materials. This finding indicates that the heterogeneous structure facilitates interfacial charge transfer by reducing resistance. Electron transfer within the heterostructure was further examined using TRPL spectroscopy (Figure S8). The average PL lifetimes of the electrons were 0.60 and 0.65 ns in TpTt and MIL‐68, respectively, while the lifetimes of MIL@COF can be reached to 1.14 ns. In addition, as shown in the photocurrent intensity analysis in Figure 3i, MIL@COF exhibits the strongest photocurrent response, indicating a higher current density. All of this indicates that the heterojunction formed between MIL‐68 and COF, which facilitates more efficient separation of photogenerated carriers [35, 36, 37].

**FIGURE 4:**
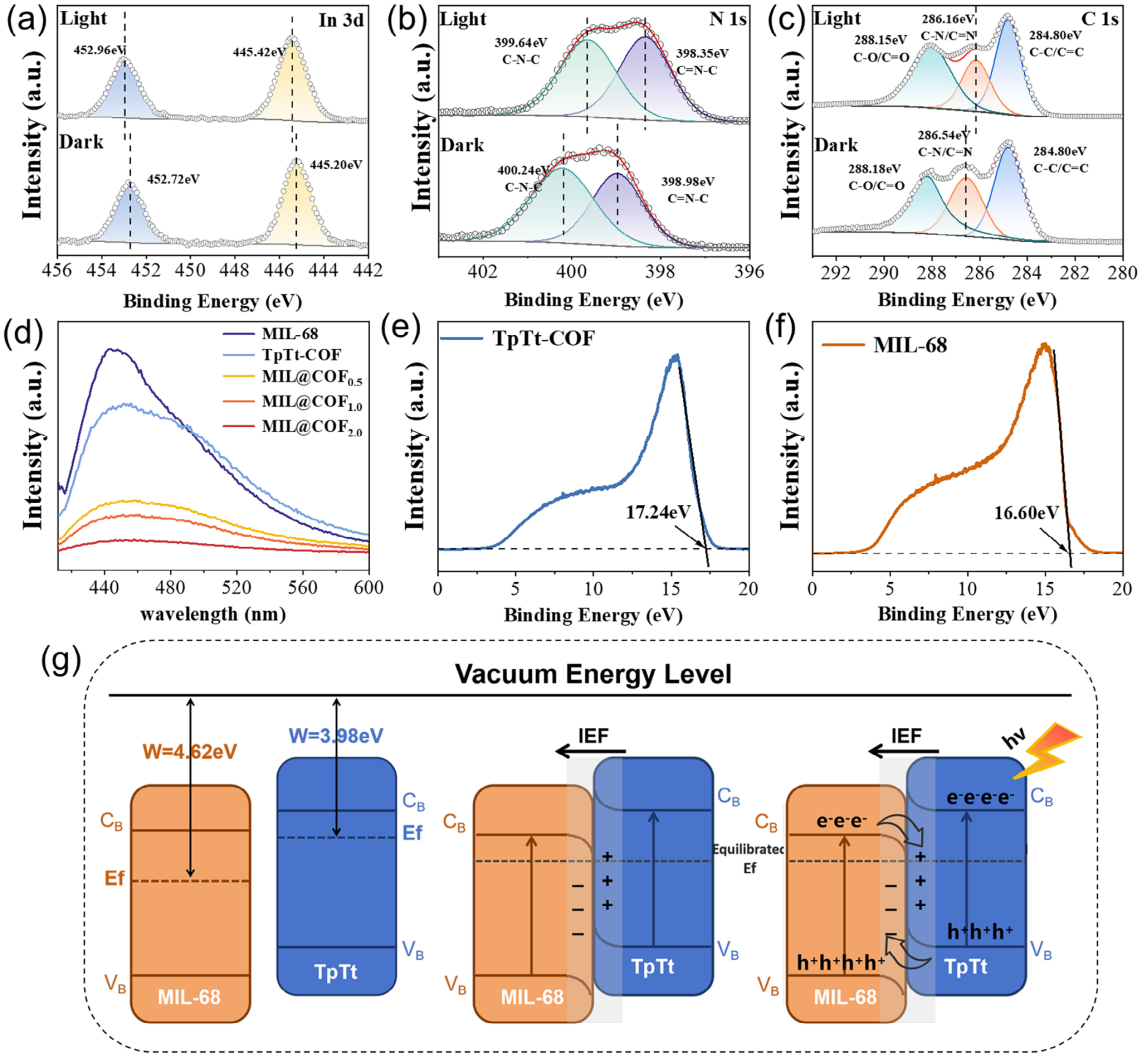
XPS measurements, in situ high‐resolution XPS spectra of (a) In 3d, (b) N 1s, and (c) C 1s of MIL@COF before and after illumination. (d) PL spactra of MIL‐68, TpTt‐COF, and MIL@COF, Ultraviolet photoelectron spectroscopy of (e) TpTt‐COF and (f) MIL‐68. (g) Schematic representation of the formation of IEF by MIL@COF in the light.

To further elucidate the electron transfer behavior between MIL‐68 and TpTt‐COF within MIL@COF, high‐resolution XPS spectra of representative elements were analyzed. As illustrated in Figure 4a, the high‐resolution In 3d XPS spectrum exhibits two distinct peaks at 445.75 and 452.75 eV, corresponding to In 3d_5/2_ and In 3d_3/2_ peaks, respectively, which arise from spin‐orbit splitting [38]. The high‐resolution N 1s spectrum (Figure 4b) is fitted into two peaks at 398.6 and 400.0 eV, corresponding to triazine C═N─C bonds and amide‐ or amine‐linked C─N bonds, respectively [39]. The high‐resolution C 1s spectrum (Figure 4c) is deconvoluted into three peaks at 284.80, 286.54, and 288.18 eV, corresponding to C─C/C═C, C─N/C═N, and C═O/C─O functional groups, respectively [40]. The XPS spectra of MIL@COF under dark and illuminated conditions are compared in the diagram. Under illumination, the binding energies of In 3d_5/2_ and In 3d_3/2_ peaks shift positively by approximately 0.22 and 0.24 eV, respectively, indicating electron transfer away from MIL‐68 to TpTt‐COF, consistent with the formation of an internal electric field at the heterojunction interface. Under illumination, the high‐resolution N 1s spectrum shows a negative shift of approximately 0.6 eV in the peaks corresponding to triazine‐based C─N═C bond. This finding is further supported by a similar negative shift observed for C─N/C═N peaks in the C 1s spectrum, confirming the migration of electrons from MIL‐68 to TpTt‐COF. This electron migration significantly increases electron accumulation within the conduction band of TpTt‐COF, thereby enhancing its photocatalytic activity [41]. These findings collectively confirm the formation of an internal electric field and directional electron transfer from MIL‐68 to TpTt‐COF under illumination, which is consistent with the S‐scheme heterojunction mechanism proposed above.

Meanwhile, the transfer and utilization of photogenerated electrons in MIL@COF were investigated through photoelectrochemical analysis. The PL spectrum of the material is presented in Figure 4d. Under an excitation wavelength of 375 nm, the corresponding PL emission peaks appear at 437, 449, 446, 447, and 451 nm, respectively. The PL intensity of MIL@COF is significantly lower than that of single‐component MIL‐68 and TpTt, indicating that the formation of the heterojunction effectively suppresses the recombination of photogenerated carriers [42]. This suppression enhances charge separation efficiency, thereby improving photocatalytic activity. This suggests that the structure of MIL@COF facilitates the radial migration of photogenerated electrons, directing them toward the surface. This process effectively suppresses electron‐hole recombination, thereby enhancing charge separation efficiency [42].

As depicted in Figure 4e,f, the secondary electron cutoff edge (E_cutoff_) derived from ultraviolet photoelectron spectroscopy (UPS, He I = 21.22 eV) reveals work functions of MIL‐68 and TpTt‐COF as 4.62 and 3.98 eV relative to the vacuum level [43]. Integrating the energy band coordinates determined by UV‐vis and Mott‐Schottky analyses, Figure 4g illustrates the band structure evolution of the two porous materials before and after interfacial contact. The interfacial contact induces upward band bending in TpTt‐COF and downward bending in MIL‐68 through Fermi level (Ef) alignment, characteristic of an S‐scheme heterojunction configuration. Concurrently, interfacial electron transfer occurs from TpTt‐COF to MIL‐68 owing to the latter's lower Fermi level. This results in electron‐deficient (positively charged) TpTt‐COF and electron‐enriched (negatively charged) MIL‐68 at the interface. The synergistic effects of band bending and charge density disparity generate an intrinsic electric field (IEF) oriented from TpTt‐COF toward MIL‐68 at the interface [44]. Photoexcitation activates IEF to propel photogenerated electrons from MIL‐68's CB to TpTt‐COF's VB. The combined band alignment and IEF further inhibit undesirable electron‐hole recombination pathways: electrons in TpTt's CB to MIL‐68's CB, and holes from MIL‐68's VB to TpTt's VB. Such spatial charge separation significantly elevates the population of photogenerated electrons in TpTt's CB and holes retained in MIL‐68's VB [45]. The MIL@COF composite therefore exhibits superior redox performance.

### 2.3 Carrier Dynamics and Dual‐Channel Transmission Mechanism

**FIGURE 5:**
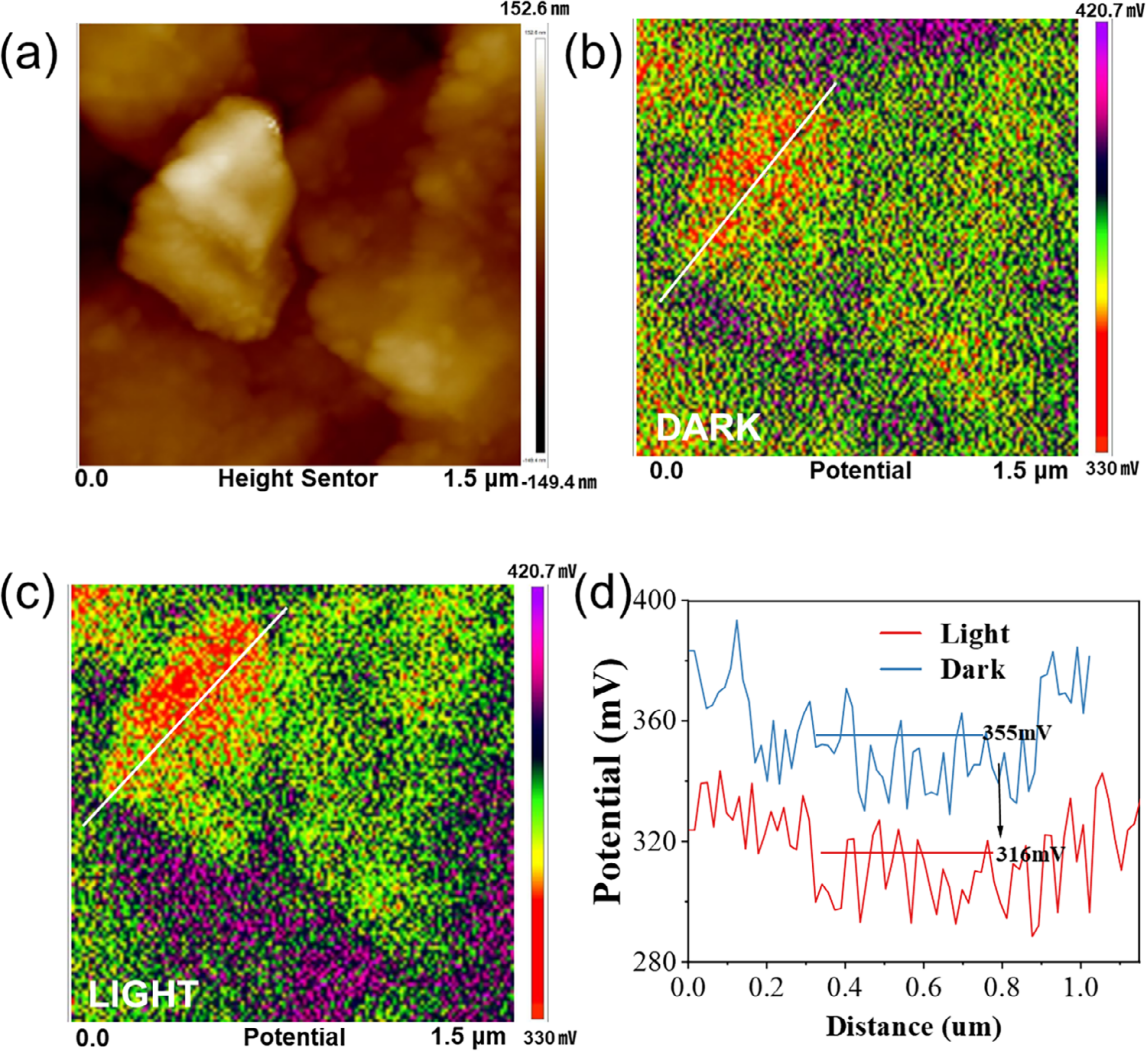
(a) KPFM morphology of MIL@COF and the corresponding MIL@COF; (b) SP maps in the dark and (c) under light irradiation; (d) SP images of cross sections.

To gain deeper insight into the spatial charge separation behavior of photogenerated carriers in MIL@COF under illumination, Kelvin probe force microscope (KPFM) scanning was performed over a 1.5 × 1.5 µm region on the material surface (Figure 5a). Figure 5b,c illustrates the potential mapping images of MIL@COF under dark and illuminated conditions. As depicted, the heterojunction demonstrates a relatively high electric field intensity even in the absence of light, while the built‐in electric field induced by the S‐scheme serves as a potent driving force for charge transfer [46]. Under illumination, the material's average surface potential shows a decreasing trend. Figure 5d depicts the potential distribution curve along the marked region and the calculated surface potential difference (△SP). As illustrated, the △SP of the MIL@COF drops by about 39 mV upon light exposure. This validates the efficient reverse transfer of photoexcited electrons and the spatial separation of photogenerated charge carriers [47], further substantiating the high electron‐hole separation efficiency of the composite.

**FIGURE 6:**
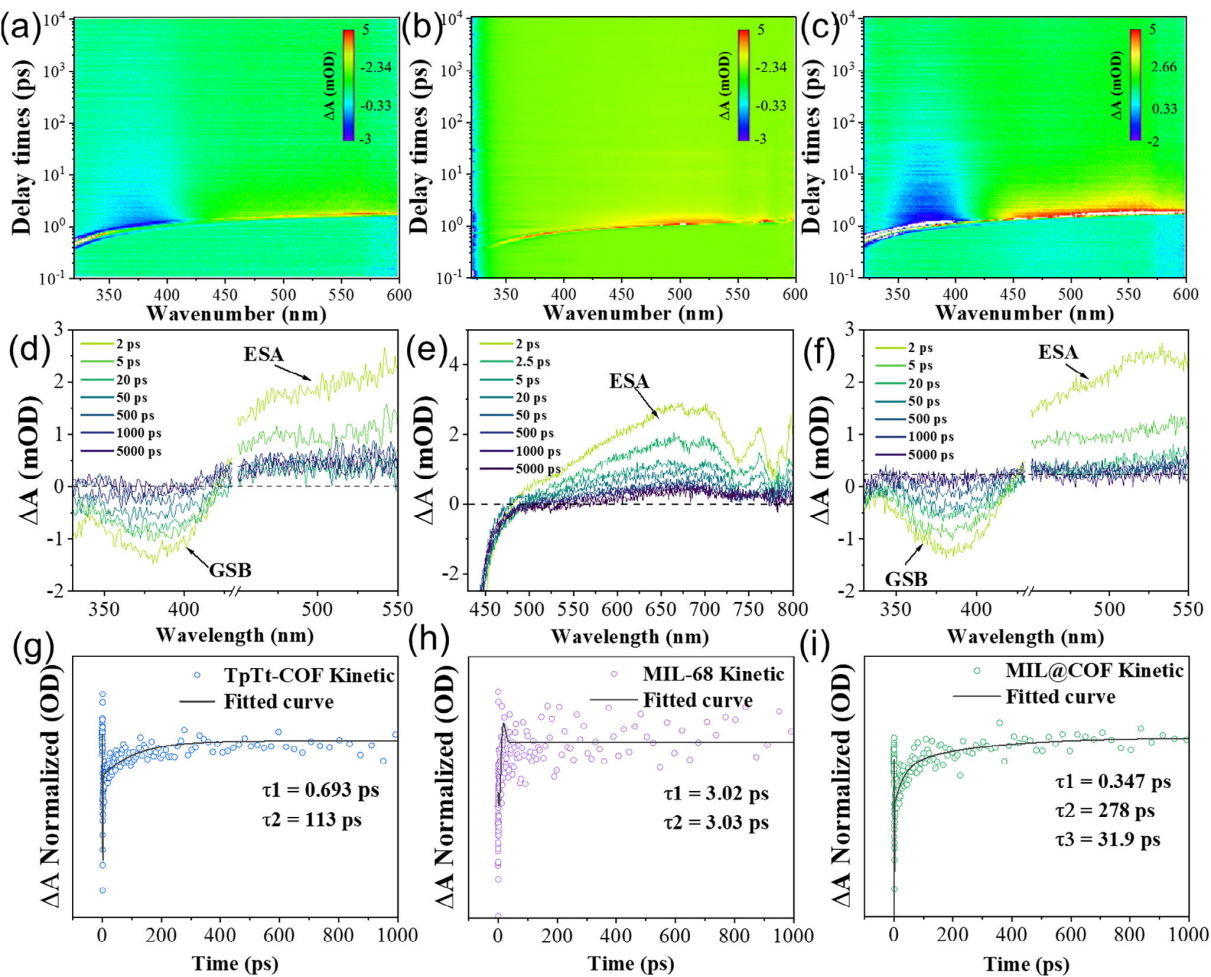
(a,d) TpTt‐COF, (b,e) MIL‐68, and (c,f) MIL@COF 2D mapping of the TAS signal on ps—ns time scales. (g) TpTt‐COF and (i) MIL@COF at 400 nm and (h) MIL‐68 at 675 nm (Embedding Scheme: Decay Path of Photogenerated Electrons) Normalized decay kinetic curves.

In order to further investigate the carrier transfer mechanism, the kinetic process of photogenerated charge carriers was analyzed by fs‐TAS at 320 nm pump pulse. A clear negative signal at 380 nm in the fs‐TAS spectrum of TpTt‐COF is attributed to its ground state bleaching (GSB) signal (Figure 6a,d). The fs‐TAS spectrum of MIL‐68 exhibits strong fluorescence in the UV band. Therefore, only the visible band TA spectrum was examined. A broad positive peak spanning 480 to 780 nm was observed, signifying an ESA signal attributed to further excitation of excited‐state electrons (Figure 6b,e). The spectra of the parent materials were first analyzed, and the ESA signals of TpTt‐COF showed a phenomenon of decay followed by recovery in the delay time range, which could be attributed to the presence of dynamically balanced excited state processes or multistep relaxation paths in the system [47]. In addition, it is found that the MIL@COF has a much longer GSB signal decay rate than that of the parent material, while the ESA signal decay rate is accelerated; the long lifetime of the GSB signal indicates that the carriers are better separated, and the photogenerated electrons and holes can exist in the material for a longer period of time, which also suggests the role of the S‐scheme structure in enhancing the separation efficiency of photogenerated carriers (Figure 6c,f). The rate of ESA signal attenuation has increased. It is speculated that oxygen vacancies have captured some photogenerated electrons, thereby suppressing the relaxation of excited‐state electrons and the recombination of photogenerated electrons and holes [48, 49]. In addition, in the delay time range, we found a rising trend at 380 nm in the TA spectrum of MIL@COF while the ESA signal shifted in the long range of the 500 nm band, suggesting that the exciton produced new matter during the decay process [50]. This result indicates the generation of internal charge separated (i‐CS) states in MIL@COF. The generation of this signal indicates the rapid separation of photogenerated excitons (electron‐hole pairs) into free electrons and holes inside the material, which allows MIL@COF to exhibit superior performance in photocatalysis [51].

The decay of TpTt‐COF at 400 nm was fitted with a single exponential model (Figure 6g), revealing two relaxation pathways for photogenerated electrons. The short lifetime (τ0 = 1.05 ps) is attributed to lattice electron diffusion [52], while the long lifetime (τ1 = 105 ps) is not due to electron‐hole recombination [53], which contradicts the transient photoluminescence (TRPL 0.6 ns) data. Therefore, this long lifetime process should be attributed to the recombination of photogenerated electrons with trap holes. Meanwhile, as shown in Figure 6h, since only the ESA signal of MIL‐68 could be detected, the decay at 675 nm was fitted with a single exponential model, where τ0 = 1.38 ps is attributed to electron diffusion and τ1 = 12.1 ps is attributed to the recombination of photogenerated electrons with trap holes [54]. For the GSB decay signal of MIL@COF at 400 nm, it can be fitted with a three‐exponential model (Figure 6i), indicating three relaxation pathways. The short time τ0 = 1.42 ps is attributed to the diffusion of electrons within the lattice, which is consistent with the τ0 of the above two materials. The relatively short lifetime τ1 = 2.7 ps is much lower than the τ1 of the parent materials, so it is speculated that it is not radiative recombination but rather the capture of electrons from the conduction band minimum (CBM) of MIL‐68 to defect‐induced trap levels; another fitted lifetime τ2 = 23.4 ps is attributed to the interfacial electron transfer dominated by covalent bonds. The τ3 = 288 ps is significantly longer than the τ1 of MIL‐68 and TpTt‐COF mentioned earlier, which may be attributed to the electrons in MIL‐68 falling back to the ground state from the excited state of TpTt after interfacial transfer, resulting in a significant extension of the lifetime. It could be that the electrons captured by defect levels in MIL‐68, after being re‐excited by light, undergo radiative transitions and fall back to the ground state. However, the sum of τ0 to τ3 still contradicts the TRPL (1.4 ns), suggesting that the ground state is still trap holes as mentioned earlier. The above results have clearly illustrated the temporal sequence of charge events.

### 2.4 Photocatalytic Performance and Reaction Mechanism

**FIGURE 7:**
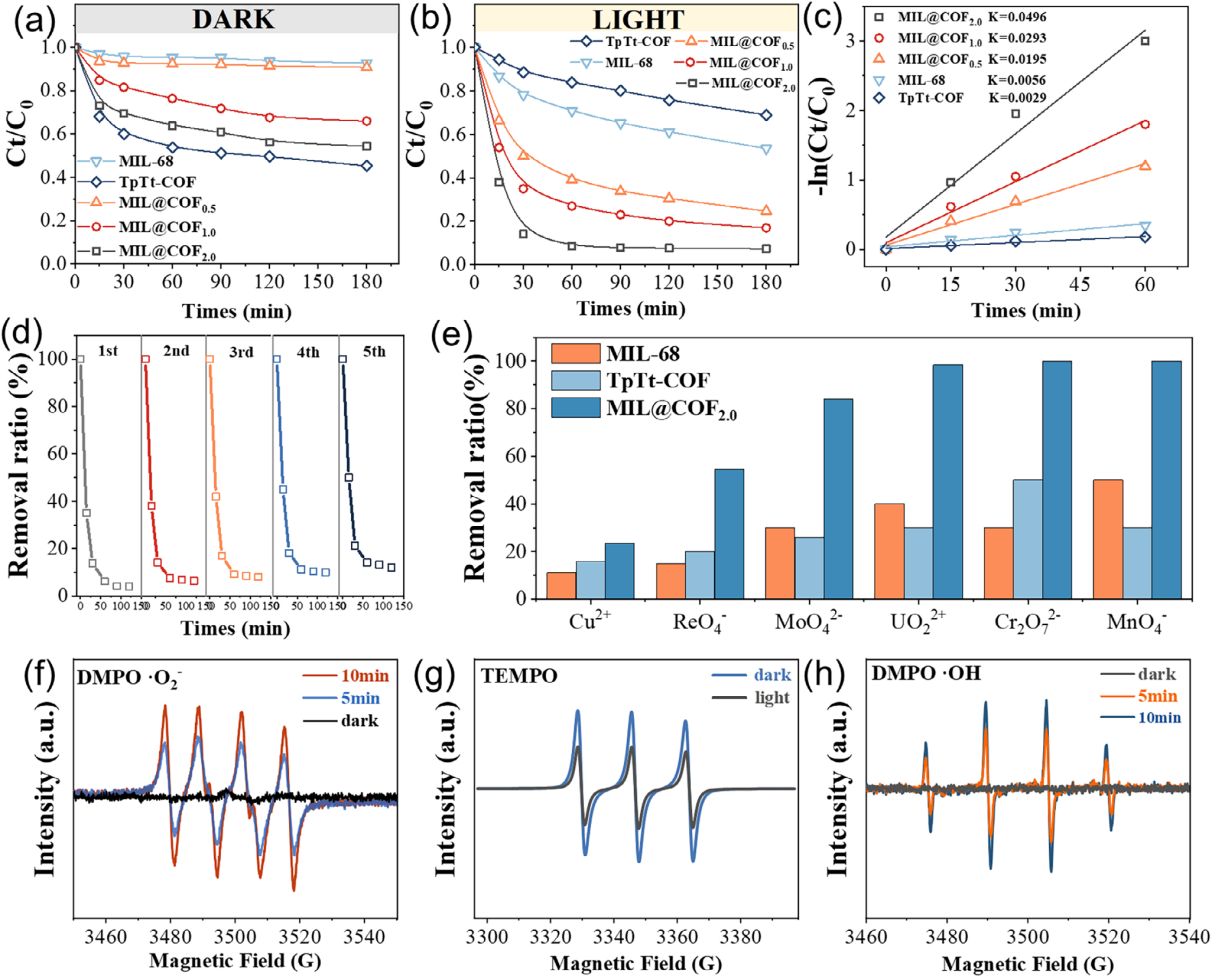
(a,b) UO_2_^2+^ removal curves under dark and light conditions, (c) linear fit and kinetic constants for photocatalytic extraction of uranium, (d) recycling performance of five, (e) the catalytic removal efficiency of various heavy metal ions, the active substances in the catalytic process; (f) EPR spectra of e–TEMPO, (g) ·OH^−^DMPO, and (h) ·O_2_^−^DMPO.

To evaluate the catalytic removal efficiency for UO_2_^2+^, tests were conducted without sacrificial agents, using an initial uranium concentration of 100 ppm, solid‐liquid ratio of 0.4 g L^−1^, and pH = 5. As shown in Figure 7a,b, pristine MIL‐68 and TpTt‐COF exhibit limited UO_2_^2+^ removal due to poor charge separation. In contrast, MIL@COF composites shows significantly enhanced performance. Quasi‐first‐order kinetics give rate constants of 0.0056 min^−1^ for MIL‐68, 0.0029 min^−1^ for TpTt‐COF, and 0.0496 min^−1^ for MIL@COF_2.0,_ which shows that 8.8 and 17.1 times higher than the parent materials, respectively (Figure 7c). The composite achieved 97.6% removal within 60 min, with completion in 30 min. Compared to a physical mixture (38% removal in 180 min, Figure S9), MIL@COF reached 98% in 60 min and 99.2% at 180 min, confirming that heterojunction formation promotes charge separation and drastically improves catalytic efficiency. Furthermore, after five cycles, the material still maintained extremely high removal efficiency (Figure 7d), that indicates composite obtain a good stability.

To demonstrate the broad‐spectrum characteristics of the catalysts in heavy metal pollution control, its efficacy in removing various heavy metal ions from aqueous solution was systematically investigated, as illustrated in Figure 7e. The removal efficiencies for key ions including Re, Mo, U, Cr, and Mn were all found to exceed 60%, demonstrating the material's broad‐spectrum remediation capability. Remarkably, complete removal—achieving 100% efficiency—was observed for both Cr and Mn ions, highlighting the particular affinity and high effectiveness of the material toward these contaminants. Furthermore, in comparison to its individual components, the MIL@COF composite exhibits significantly enhanced photocatalytic removal performance, substantially outperforming both pure MIL‐68 and TpTt‐COF under identical conditions. This pronounced improvement underscores the synergistic effect arising from the composite formation, which facilitates more efficient charge separation and enhanced surface reactivity.

The removal efficiency of composites for UO_2_^2+^ under different environmental conditions was systematically evaluated. As shown in Figure S10, degradation efficiency exceeded 90% within pH 4–7, but decreased gradually from pH 3 to 4, due to protonation‐induced surface positive charging under acidic conditions [55]. Furthermore, the material also maintained high UO_2_^2+^ removal in the presence of competing anions (NO_3_^−^, Cl^−^, CO_3_^2−^, SO_4_^2−^, ClO_4_^−^, F^−^). Even under interference from various metal cations, MIL@COF achieves over 97% removal (Figure S11), demonstrating exceptional ion tolerance. Active species tests (Figure S12) revealed that adding PBQ, AgNO_3_, or IPA significantly inhibited UO_2_^2+^ removal, confirming that photogenerated e^−^, ·OH and ·O_2_^−^ are key active species in the catalytic process.

To gain a deeper understanding of the types of active substances involved in the catalytic process, electron paramagnetic resonance (EPR) analysis was performed on substances with redox activity during the photocatalytic process (Figure 7f–h). There was no ·OH signals detected under dark conditions, while a certain amount of vacancy signals was detected when collecting ·O_2_^−^ signals. After exposure to xenon light, the EPR spectra exhibited strong ·OH and ·O_2_^−^ signals. This confirms the presence of ·OH and ·O_2_^−^ in the photocatalytic process, which may be important active species in the reaction process. The intensity of the TEMPO signal decreases with prolonged irradiation time, which is attributed to the binding of photo‐generated electrons with TEMPO, indicating that under irradiation conditions, a large number of e^−^ migrate to the material surface and reduce the active species adsorbed on the material surface [56].

Totally, MIL@COF exhibits exceptional photocatalytic efficiency. Its activity, significantly surpassing parental materials, stems from enhanced charge separation via heterojunction formation. The composite demonstrates broad‐spectrum heavy metal removal, high tolerance to ionic interferents, and maintains efficacy across a wide pH range, indicating strong potential for practical wastewater treatment.

## 3 Theoretical Verification of the Dual‐Channel Mechanism

**FIGURE 8:**
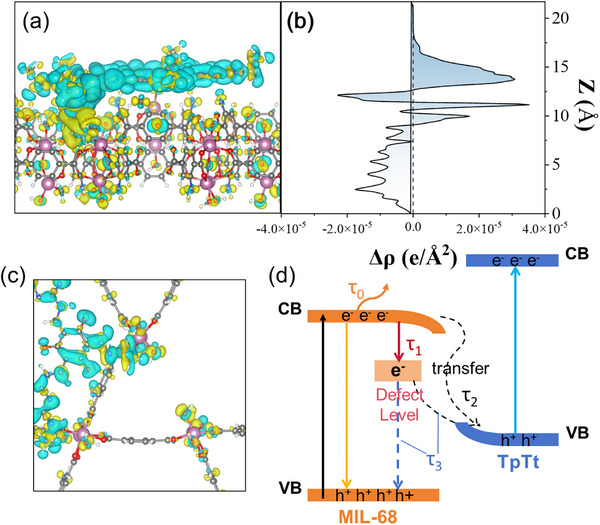
(a,c) EDD maps of MIL@COF, (b) average charge density difference, and (d) the light‐induced electron transfer and charge recombination pathways of the MIL@COF.

To provide more insights into the specific charge migration behavior of carriers at the interface, the charge density calculated using DFT was used to track the transfer of electrons, the basic model structure is shown in Figures S13,S14. Charge accumulation and depletion are shown in yellow and blue, respectively, as shown in Figure 8b. A large amount of charge transfer occurs at the interface, giving the surface TpTt‐COF a positive trend, in agreement with the experimental results. The average differential charge at the MIL@COF interface is a function of z [57], as shown in Figure 8a. The electron cloud density is significantly clustered at the ‐C─N‐ bond at the interface, indicating that the strong action of bond sums and the covalent bond as a bridge for charge transfer can accelerate the electron transfer. It is also shown that electrons migrate from TpTt‐COF to MIL‐68 through the tight heterogeneous interface, and the charge density difference brought by the charge transfer at this interface induces an internal electric field in the MIL@COF, which facilitates the separation of photogenerated charge carriers. The charge density distribution at the OVs shown in Figure 8c, indicates that the OVs is electrically neutral overall, which is consistent with theory. The OVs acts as an electron trap, effectively capturing free electrons from the conduction band. This charge localization phenomenon explains the trap effect of the OVs. Since OVs introduce local electron acceptor states, electrons are attracted and captured onto the defect energy levels, thereby preventing their free migration within the material [51]. This process significantly reduces electron mobility, making OVs an effective electron capture center.

In this study, the introduction of OVs in S‐scheme was found to effectively establish new electron migration pathways, significantly enhancing photocatalytic performance. OVs act as electron traps, forming defect energy levels within the material's bandgap, preferentially capturing and trapping photo‐generated electrons, thereby triggering the reconfiguration of interfacial charge behavior (Figure 8d). Notably, this process induces captured electrons to traverse the heterojunction barrier via defect‐assisted pathways, greatly promoting the consumption of low‐energy carriers while retaining and enriching photogenerated electrons with strong reducing ability at catalytic active sites. This mechanism not only significantly suppresses charge recombination in the bulk and at interfaces but also optimizes surface reaction kinetics, thereby enhancing the overall efficiency of catalytic reduction reactions. Therefore, OVs engineering provides a reliable strategy for achieving synergistic improvements in charge separation and utilization efficiency in S‐scheme by establishing efficient electron transport pathways.

## 4 Conclusion

In this work, the OVs‐mediated MOF/COF S‐scheme has been successfully constructed through the strategy of vacuum‐induced covalent bonding, which solves the difficulties of inefficient carrier separation and a single charge transport pathway in conventional heterojunctions, and realizes the extensive removal of heavy metal pollutants in water bodies. The main points of this work are (1) the heterojunction with covalent bonding and realizes a lattice‐matched and continuous S‐scheme, which constructs a built‐in electric field and enhances the spatial separation efficiency of the photocarriers, and (2) the OVs triggers an additional carrier migration mechanism through the localized defect state, which prolongs the carrier lifetime up to 1.6 ps. Synchrotron radiation X‐ray absorption spectroscopy (XAFS) and femtosecond transient spectroscopy (fs‐TAS) confirm the construction of OVs and their synergistic effect with S‐sheme. Theoretical calculations show that the difference in interfacial charge and the charge departure induced by OVs synergistically optimize the reduction kinetics of heavy metal ions. The “interface‐defect dual regulation” strategy proposed in this study provides a new paradigm for light‐driven heavy metal pollution control, and the synthesis method of vacuum thermally induced OVs can be extended to the design of other porous heterojunction systems, which will promote the precise regulation of environmental remediation technologies.

## 5 Supporting Information

Supporting Information is available from the Wiley Online Library or from the author.

## Funding

The National Natural Science Foundation of China (Grant Nos. 22276193, 12075066, 12205056, and U21B2094).

## Conflicts of Interest

The authors declare no conflict of interest.

## Supporting information




**Supporting File**: advs73496‐sup‐0001‐SuppMat. docx.

## Data Availability

The data that support the findings of this study are available from the corresponding author upon reasonable request.
