# Supplementary material for: Defect‐Engineered MOF/COF S‐Scheme Heterojunction With Dual‐Channel Charge Transport for Ultraefficient Solar‐Powered Heavy Metal Remediation
Source: Adv Sci (Weinh). 2026 Jan 15;13(13):e20433. doi: 10.1002/advs.202520433 (PMC12955943; doi:10.1002/advs.202520433)
Supplement: Supplementary file 1 — Supporting File: advs73496‐sup‐0001‐SuppMat. docx. [file ADVS-13-e20433-s001.docx]

**Supplementary Information**

**Defect-Engineered MOF/COF S-Scheme Heterojunction with Dual-Channel Charge Transport for Ultraefficient Solar-Powered Heavy Metal Remediation**

Yuqian Zhong, Xinpeng Wang, Weiqun Shi, and Liyong Yuan*

**Table of Contents**

**S1. Supplementary Tables**

**S2. Supplementary Figures**

**Figure S1**. SEM image of MIL-68-NH_2_

**Figure S2**. SEM image of TpTt

**Figure S3.** FI-TR of original materials and intermediates

**Figure S4.** XRD of original materials and intermediates

**Figure S5.** The percentage distribution map of various states of oxygen

**Figure S6**. The full XPS spectrum of original materials and materials.

**Figure S7.** The performance of simple mechanical mixing and covalent bond combination (removal kitnet）

**Figure S8.** The removal efficiency of uranium under different removal agent systems

**Figure S9.** FI-TR spectra before and after catalytic reactions

**Figure S10.** The removal efficiency of uranium under different pH environments

**Figure S11.** Anti-anion selectivity

**Figure S12**. DFT-optimized configuration of Heterogeneous structure

**Figure S13.** DFT-optimized configuration of Heterogeneous structure

**Figure S14.** WT-EXAFS for In K-edge of (a)MIL-68, (b)MIL@COF, (C)In foil,and (d)In_2_O_3_

## Characterizations

The FT-IR spectra were recorded using the Bruker TENSOR 27 instrument. The powder X-ray diffraction (PXRD) data of nanomaterials were collected on the Bruker AXS D8 Advance A25 powder X-ray diffractometer (40 kV, 40 mA), using Cu Kα (λ=1.5406 A) radiation, with an increment of 0.05°from 2θ = 2°to 40°. Scanning electron microscopy and high-resolution transmission electron microscopy (SEM, JEM-2010, JEOL) were adopted; HRTEM, FEI Talos F200X G2. The Kelvin probe force microscopy (KPFM) and piezoelectric microscopy (PFM) tests were conducted using atomic force microscopy (AFM) and Bruker Dimension lcon. Under Al K irradiation, X-ray photoelectron spectroscopy (XPS) was obtained using Thermo VG Multilab 2000X. The isotherms of nitrogen adsorption and desorption at 77 K were determined by the Micromeritics ASAP 2020M system. The sample was degassed at 120°C for 8 hours before measurement. The surface area of the adsorption data was determined by the Brunor-Emmett-Taylor (BET) method. The pore size distribution of porous materials was analyzed by using the non-local density functional theory (NLDFT). The thermal properties of nanomaterials were evaluated by using the STA PT1600 Linseis thermogravimetric analysis (TGA) instrument in a nitrogen atmosphere ranging from 30 to 800°C with a heating rate of 10°C/min. The fluorescence spectra were recorded on the fluorescence spectrophotometer (F-7000, Hitachi). Time-resolved spectra were recorded using the FLS1000 instrument. The ultraviolet/visible diffuse reflectance spectra (DRS) were recorded at room temperature using the PE Lambda 900 ultraviolet/visible spectrophotometer. The concentration of metal ions was determined by iCAP Q inductively coupled plasma mass spectrometry (ICP-MS, Thermo Fisher Scientific, USA).

## Experimental

### 2.1 Synthesis of NH2-MIL-68

First, 218.1 mg (1.2 mmol) of 2-aminoterephthalic acid (NH₂-BDC) and 432.6 mg (1.35 mmol) of indium nitrate hydrate (In(NO₃)₃·H₂O) were dissolved in 5 mL of dimethylformamide (DMF) under ultrasonication for 5 minutes. The resulting dark brown solution was transferred into a 10 mL polytetrafluoroethylene (PTFE) reactor and heated at 100°C for 24 hours. After the reaction, the precipitate was isolated by centrifugation and washed three times sequentially with anhydrous ethanol and DMF via solvent exchange. The product was then dried at 60°C for 12 hours to yield NH₂-MIL-68 as a pale yellow powder.

### 2.2 Synthesis of TpTt

First, 38 mg (0.3 mmol) of melamine (Tt) and 63 mg (0.3 mmol) of triacetaldehyde interphenol (Tp) were dispersed in a mixed solvent system containing 2 mL of N,N-dimethylacetamide (DMAc) and 1 mL of dimethyl sulfoxide (DMSO) (2:1, v/v). The mixture was sonicated for 15 minutes to ensure homogeneous dispersion, followed by the addition of 0.3 mL of 6 M glacial acetic acid (HAc) as a catalyst. The reaction mixture underwent three freeze-pump-thaw cycles to degas the system, after which the reaction vessel was sealed under vacuum and heated at 120°C for 72 hours. The precipitated product was collected by filtration, washed repeatedly with DMAc to remove residual starting materials, and treated with excess acetone. Finally, the yellowish powder was dried under vacuum at 100°C for 12 hours to afford the synthesized **TpTt-COF** as a crystalline solid.

### 2.3 Synthesis of MIL-CHO

First, 500 mg of Tp was dissolved in a round-bottomed flask containing a mixed solvent of N,N-dimethylacetamide (DMAc, 66 mL) and dimethyl sulfoxide (DMSO, 34 mL) (66:34, v/v) under continuous stirring. To this Tp-containing solution, 1 g of NH₂-MIL-68 was added, and the mixture was sonicated for 15 minutes to achieve a homogeneous suspension. Subsequently, 6 mL of 6 M acetic acid (HAc) was introduced into the suspension, and the reaction system was sealed and heated at 80°C for 24 hours. The resulting precipitate was isolated by centrifugation, washed sequentially with DMAc and acetone to eliminate unreacted precursors or byproducts, and finally dried under vacuum at 80°C for 12 hours to yield **Tp-modified MIL-CHO** as a white powder.

### 2.4 Synthesis of MIL@COF

To synthesize MIL@COF composites with varying COF loadings (0.5, 1.0, and 2.0), three separate reactions were conducted. For each reaction, 180 mg of MIL-CHO was combined with triacetaldehyde mesophenol (Tp) and melamine (Tt) at distinct mass ratios: **MIL@COF_0.5_**: 21 mg Tp + 12.6 mg Tt, **MIL@COF_1.0_**: 42 mg Tp + 25.3 mg Tt, **MIL@COF_2.0_**: 84 mg Tp + 50.6 mg Tt. Each mixture was added to a 20 mL Pyrex tube containing a solvent system of N,N-dimethylacetamide (DMAc, 4 mL) and dimethyl sulfoxide (DMSO, 2 mL) (2:1, v/v). The suspensions were sonicated for 15 minutes to ensure homogeneity, followed by the addition of 0.6 mL of 6 M glacial acetic acid (HAc). The tubes were then subjected to three freeze-pump-thaw cycles to degas the system, sealed under vacuum, and heated at 120°C with continuous stirring for 72 hours. Post-reaction, the precipitates were collected by centrifugation, washed extensively with acetone to remove unreacted species, and vacuum-dried at 80°C for 12 hours. The final products—**MIL@COF_0.5_**, **MIL@COF_1.0_**, and **MIL@COF_2.0_**—were obtained as light-brown powders.

**2.5 Photoelectrochemical test**

The photoelectric performance test was performed on a standard three-electrode system electrochemical workstation (Versa STAT 4, Princeton, USA). The test electrolyte was 0.5 M Na_2_SO_4_ aqueous solution. The light source was a 300 W xenon lamp with a cut-off filter. The platinum plate and the saturated Ag/AgCl electrode were used as the counter electrode and the reference electrode, respectively. The preparation method of the working electrode is as follows: an appropriate amount of sample was ground with 500 μL Nafion (5%) aqueous solution and ethanol, respectively, and then coated on the FTO conductive glass. The coating area was 1mm × 1mm. When the test voltage was 0.4 V, the photocurrent intensity of the prepared electrode was measured by turning on and off the light. EIS was measured in the frequency range of 0.1-105 Hz at open circuit voltage. According to the Mott-Schottky diagram, the flat band potential of the sample can be calculated according to E (NHE) = E (Ag/AgCl) + Eθ (Ag/AgCl), where Eθ (Ag/AgCl) = 0.197 V.

**Kubelka-Munk equation:**

$$\left( \alpha hv \right)^{n}=A\left( hv-E_{g} \right)$$

where α is the absorption coefficient, h is the Planck constant, v is the frequency of light, A is a physical quantity related to the material, Eg is the band gap energy value, and a is the absorbance. n is 1/2 for indirect band gap semiconductors and 2 for direct band gap semiconductors. Taking (αhv)n as the ordinate and hv as the abscissa, the linear section is fitted to obtain a linear equation, which is the size of the band gap energy in the x-axis intercept.

**Details of TCSPC measurements.** The TCSPC decay curves of the samples were obtained at 430 excitation wavelengths based on a time-resolved fluorescence spectrometer (TRPL) (FL3C-111 TCSPC, Horiba, Japan). The average life (t) expectancy is fitted by a triple exponential formula:

$$t=\frac{B_{1}t_{1}^{2}+B_{2}t_{2}^{2}+B_{3}t_{3}^{2}}{B_{1}t_{1}+B_{2}t_{2}+B_{3}t_{3}}$$

**2.5 Photoelectrochemical test**

## Computational details.

All DFT calculations were carried out using the CP2K code.[^1^](#_ENREF_1) All calculations employed a mixed Gaussian and planewave basis sets. Core electrons were represented with norm-conserving Goedecker-Teter-Hutter pseudopotentials,[^2-4^](#_ENREF_2) and the valence electron wave function was expanded in a double-zeta basis set with polarization functions[^5^](#_ENREF_5) along with an auxiliary plane wave basis set with an energy cutoff of 450 Ry. The generalized gradient approximation exchange-correlation functional of Perdew, Burke, and Enzerhof (PBE)[^6^](#_ENREF_6) was used. Each configuration was optimized with the Broyden-Fletcher-Goldfarb-Shanno (BGFS) algorithm with SCF convergence criteria of 1.0×10^-5^ au. To compensate the long-range van der Waals dispersion interaction, the DFT-D3 scheme[^7^](#_ENREF_7) with an empirical damped potential term was added into the energies obtained from exchange-correlation functional in all calculations.


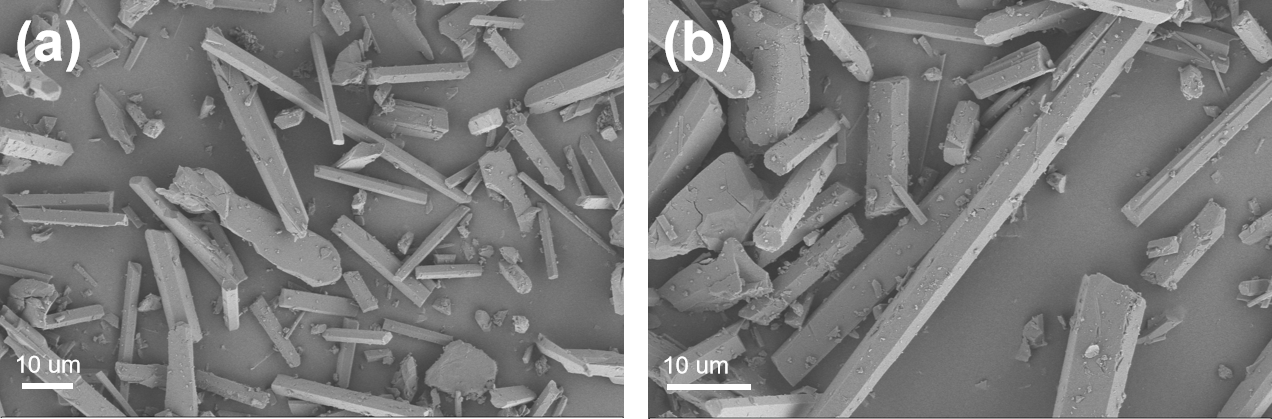


**Figure S1**. SEM image of MIL-68-NH_2_


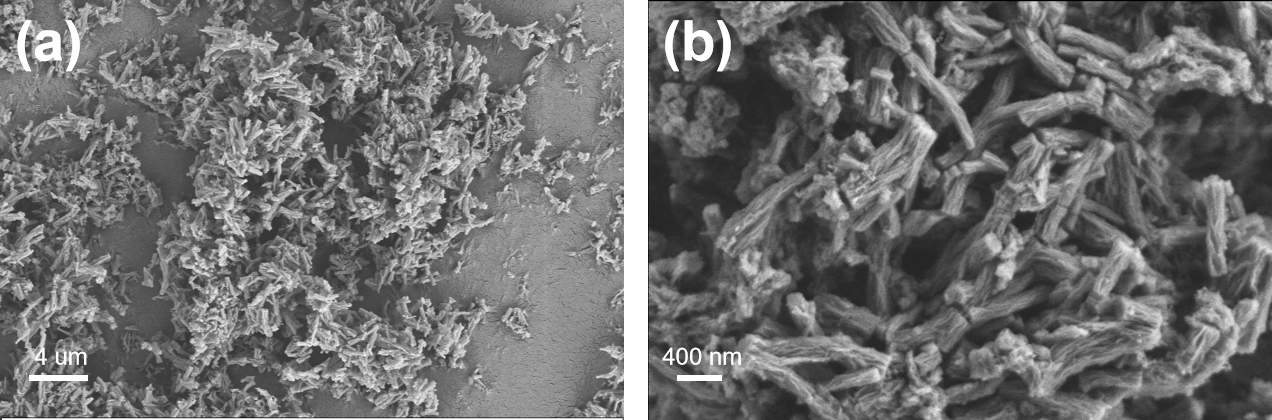


**Figure S2**. SEM image of TpTt


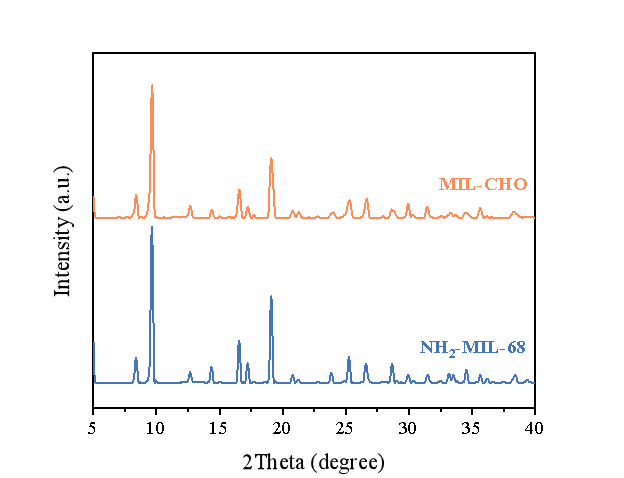


**Figure S3.** XRD of original materials and intermediates


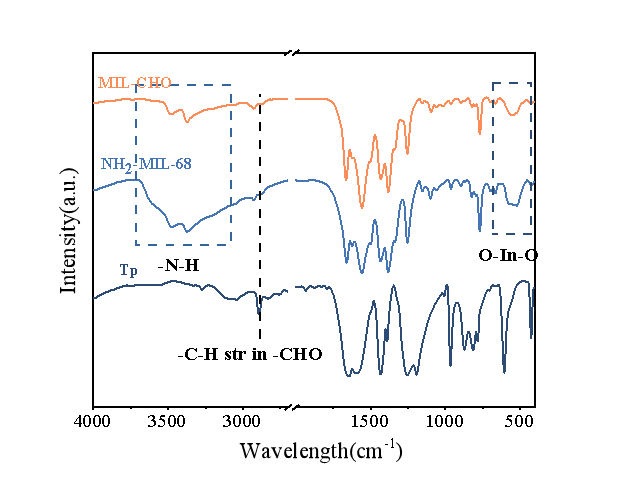


**Figure S4.** FI-TR of original materials and intermediates


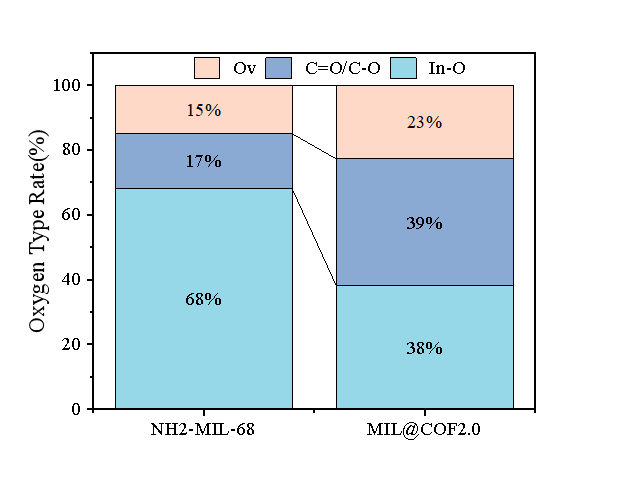


**Figure S5.** The percentage distribution map of various states of oxygen


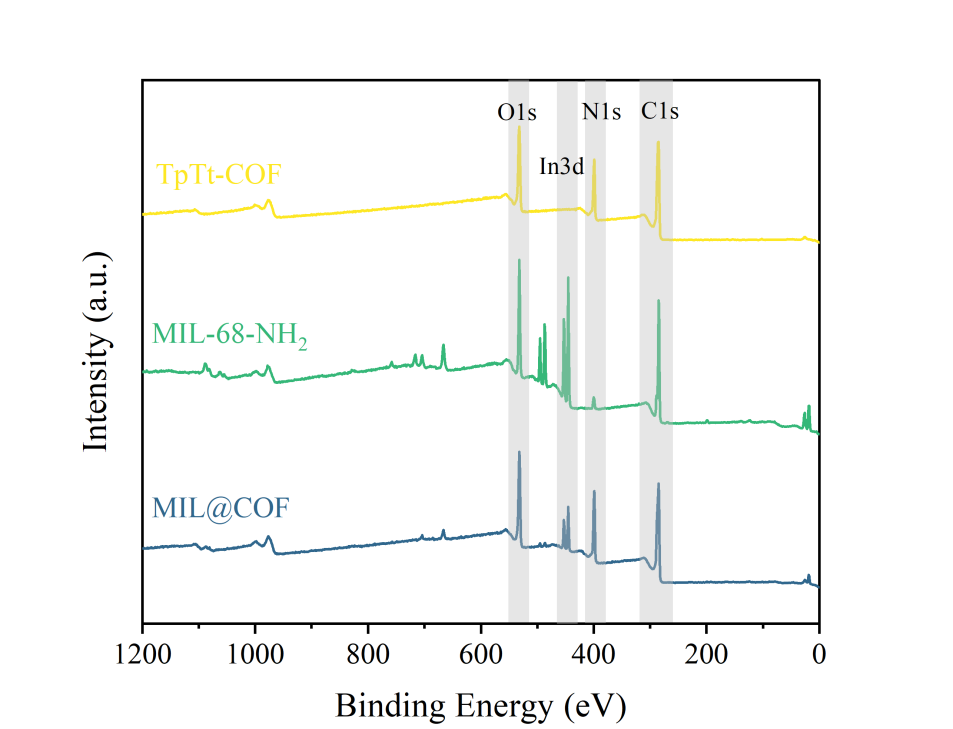


**Figure S6**. The full XPS spectrum of original materials and materials.


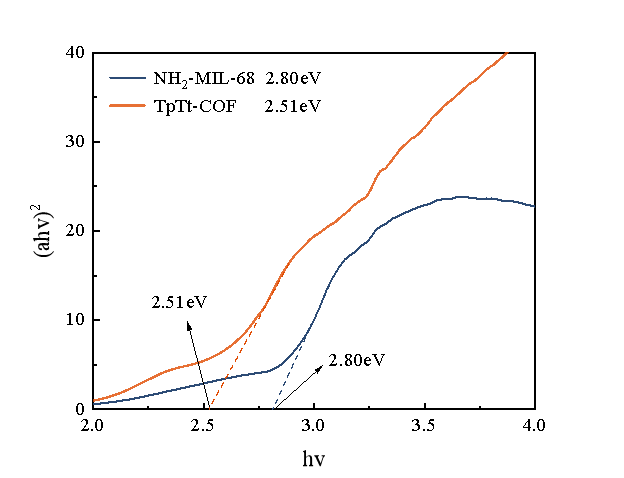


**Figure S7**. Tauc plots of UV - DRS

**Figure S8**. Time-Resolved Photoluminescence Spectroscopy)

**Figure S9.** The performance of simple mechanical mixing and covalent bond combination (removal kinetics)

**Figure S10.** The removal efficiency of uranium under different pH environments


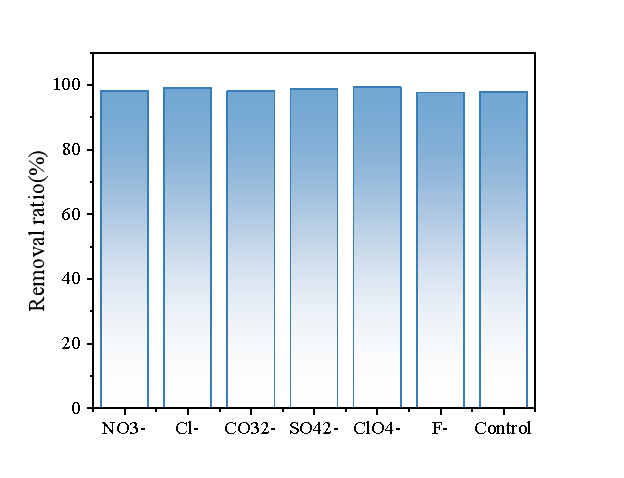


**Figure S11.** Anti-anion selectivity


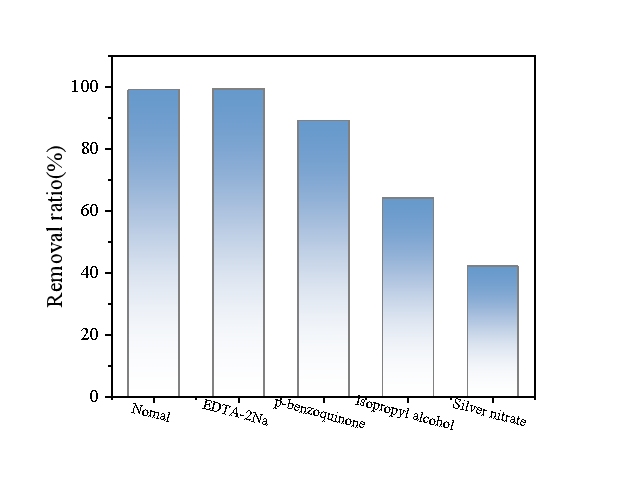


**Figure S12.** The removal efficiency of uranium under different removal agent systems


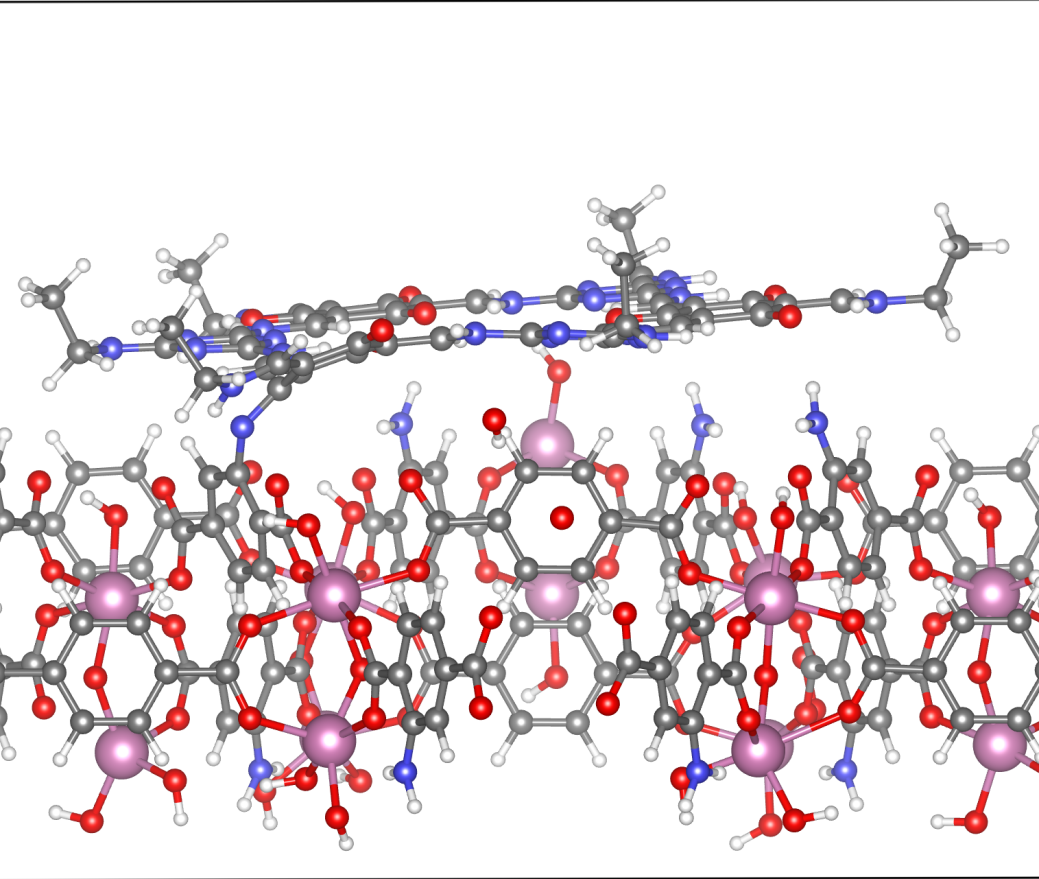


**Figure S13**. DFT-optimized configuration of Heterogeneous structure


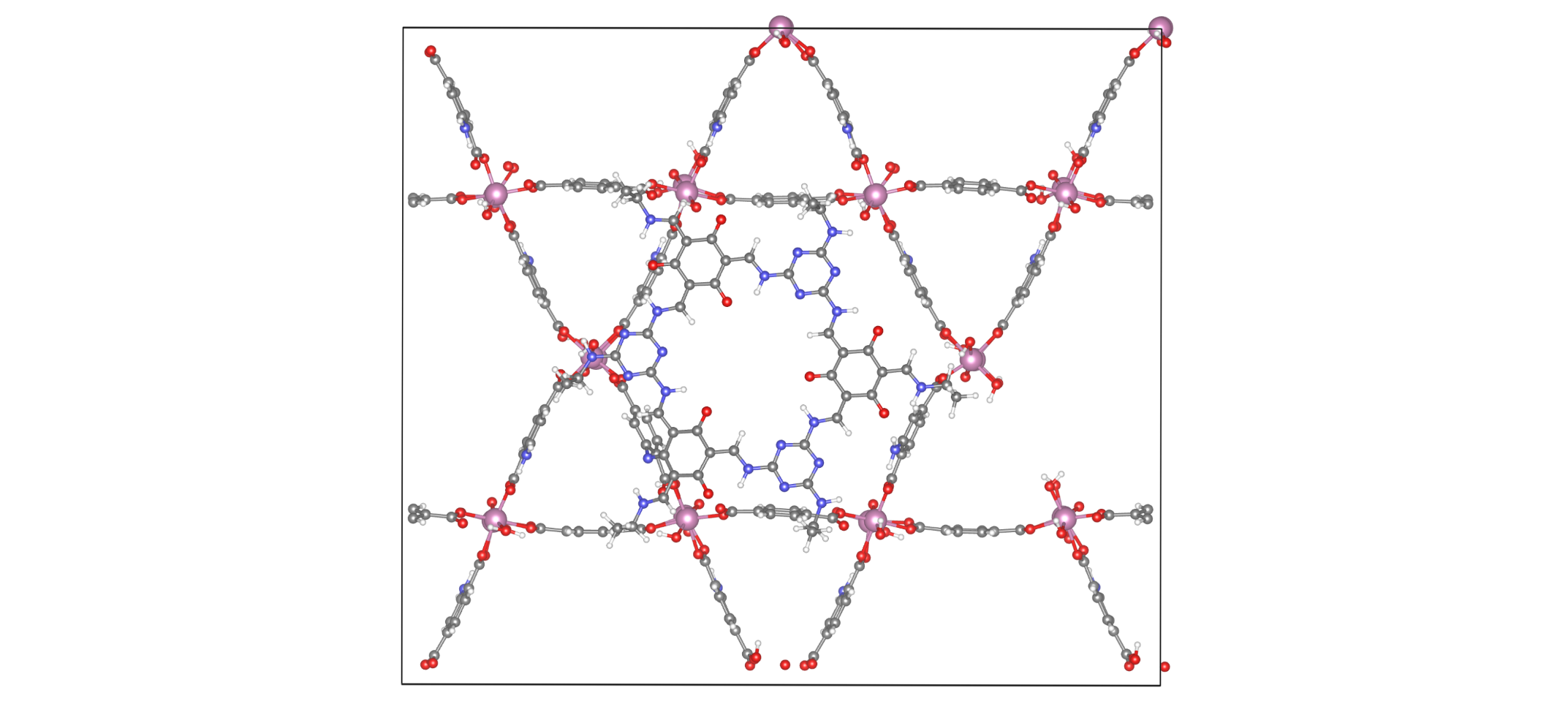


**Figure S14.** DFT-optimized configuration of Heterogeneous structure
